# Cost-Effectiveness of a Central Venous Catheter Care Bundle

**DOI:** 10.1371/journal.pone.0012815

**Published:** 2010-09-17

**Authors:** Kate A. Halton, David Cook, David L. Paterson, Nasia Safdar, Nicholas Graves

**Affiliations:** 1 The Centre for Healthcare Related Infection Surveillance and Prevention, Brisbane, Queensland, Australia; 2 Institute of Health and Biomedical Innovation, Queensland University of Technology, Brisbane, Queensland, Australia; 3 Intensive Care Unit, Princess Alexandra Hospital, Brisbane, Queensland, Australia; 4 University of Queensland Centre for Clinical Research, Royal Brisbane and Women's Hospital, Brisbane, Queensland, Australia; 5 Section of Infectious Diseases, Department of Medicine, University of Wisconsin School of Medicine and Public Health, Madison, Wisconsin, United States of America; University of Giessen Lung Center, Germany

## Abstract

**Background:**

A bundled approach to central venous catheter care is currently being promoted as an effective way of preventing catheter-related bloodstream infection (CR-BSI). Consumables used in the bundled approach are relatively inexpensive which may lead to the conclusion that the bundle is cost-effective. However, this fails to consider the nontrivial costs of the monitoring and education activities required to implement the bundle, or that alternative strategies are available to prevent CR-BSI. We evaluated the cost-effectiveness of a bundle to prevent CR-BSI in Australian intensive care patients.

**Methods and Findings:**

A Markov decision model was used to evaluate the cost-effectiveness of the bundle relative to remaining with current practice (a non-bundled approach to catheter care and uncoated catheters), or use of antimicrobial catheters. We assumed the bundle reduced relative risk of CR-BSI to 0.34. Given uncertainty about the cost of the bundle, threshold analyses were used to determine the maximum cost at which the bundle remained cost-effective relative to the other approaches to infection control. Sensitivity analyses explored how this threshold alters under different assumptions about the economic value placed on bed-days and health benefits gained by preventing infection. If clinicians are prepared to use antimicrobial catheters, the bundle is cost-effective if national 18-month implementation costs are below $1.1 million. If antimicrobial catheters are not an option the bundle must cost less than $4.3 million. If decision makers are only interested in obtaining cash-savings for the unit, and place no economic value on either the bed-days or the health benefits gained through preventing infection, these cost thresholds are reduced by two-thirds.

**Conclusions:**

A catheter care bundle has the potential to be cost-effective in the Australian intensive care setting. Rather than anticipating cash-savings from this intervention, decision makers must be prepared to invest resources in infection control to see efficiency improvements.

## Introduction

Catheter-related bloodstream infections (CR-BSIs) have been shown to increase health costs and patient morbidity [1]. They are thought to be more than 50% preventable [2] and have been the target of initiatives to create safer and efficient healthcare systems [3]. They are now included amongst the list of adverse events for which, under the Deficit Reduction Act, United States hospitals will no longer receive reimbursement [4]. This legislation created an economic imperative for decision makers to identify cost-effective infection control programmes [5]. The evidence for the effectiveness of numerous single and multi-module interventions to prevent CR-BSI has been reviewed [6], [7] but there is a lack of information about the cost-effectiveness of these activities [8].

An important intervention for which there is no economic evidence is the “bundled” approach to CR-BSI prevention promoted by the Institute for Health Improvement as part of their 100k Lives Campaign [3] (www.ihi.org). Five simple interventions that have strong scientific evidence for effectiveness are combined and integrated into clinical practice as a ‘bundle’: use of optimal hand hygiene, chlorhexidine skin antisepsis, maximal barrier precautions for catheter insertion, choice of optimal insertion site, and prompt catheter removal. Implementation involves monitoring practice compliance as well as infection rates, along with healthcare worker education and development of leadership in the area of infection control. This bundle [3] is currently being promoted for use in several countries including Australia. Studies indicate that this bundle is highly effective, with some settings reducing and maintaining their infection rates at zero [9], [10].

The cost-effectiveness of the bundle compared to other infection control interventions is currently not known. Each individual component of the bundle is relatively inexpensive to implement. This has led some clinical leaders to conclude that the bundle will be cost-effective. However, these statements do not appear to consider the nontrivial costs of monitoring and the education activities required to implement a bundled approach in a new setting. These activities require significant time and resources at the outset of the intervention and at recurrent intervals and should be included when undertaking an economic evaluation of this approach.

An evaluation should also recognize other relevant interventions to prevent CR-BSI that may need to be included in the decision. There is strong evidence that antimicrobial central venous catheters (A-CVCs) are effective at reducing risk of CR-BSI [11] and the cost-effectiveness of these devices has been have been assessed in the Australian context [12]. Some clinicians are hesitant about their use, due to risk of potential side-effects [13], [14], and may not consider use of these devices an option. However, for those clinicians whose preferences include the use, or intended use of A-CVCs, they represent an additional relevant alternative.

This study looks at the cost-effectiveness of a catheter care bundle relative to current practice, defined as a non-bundled approach to central line management with use of uncoated CVCs, and to two types of A-CVC currently available to public hospitals in Australia [15]: internally coated chlorhexidine/silver sulfadiazine (CH/SSD) catheters and minocycline and rifampicin (MR) coated catheters. This provides the first data about the economic conditions under which a catheter care bundle might be cost-effective in the intensive care unit setting.

## Methods

### Ethics statement

Ethics approval for this research was granted by Queensland University of Technology (0600000379) who waived written patient consent as all data was sourced from pre-existing databases, was provided in a de-identified form and was analyzed anonymously.

### Development of the economic model

**Figure 1:**
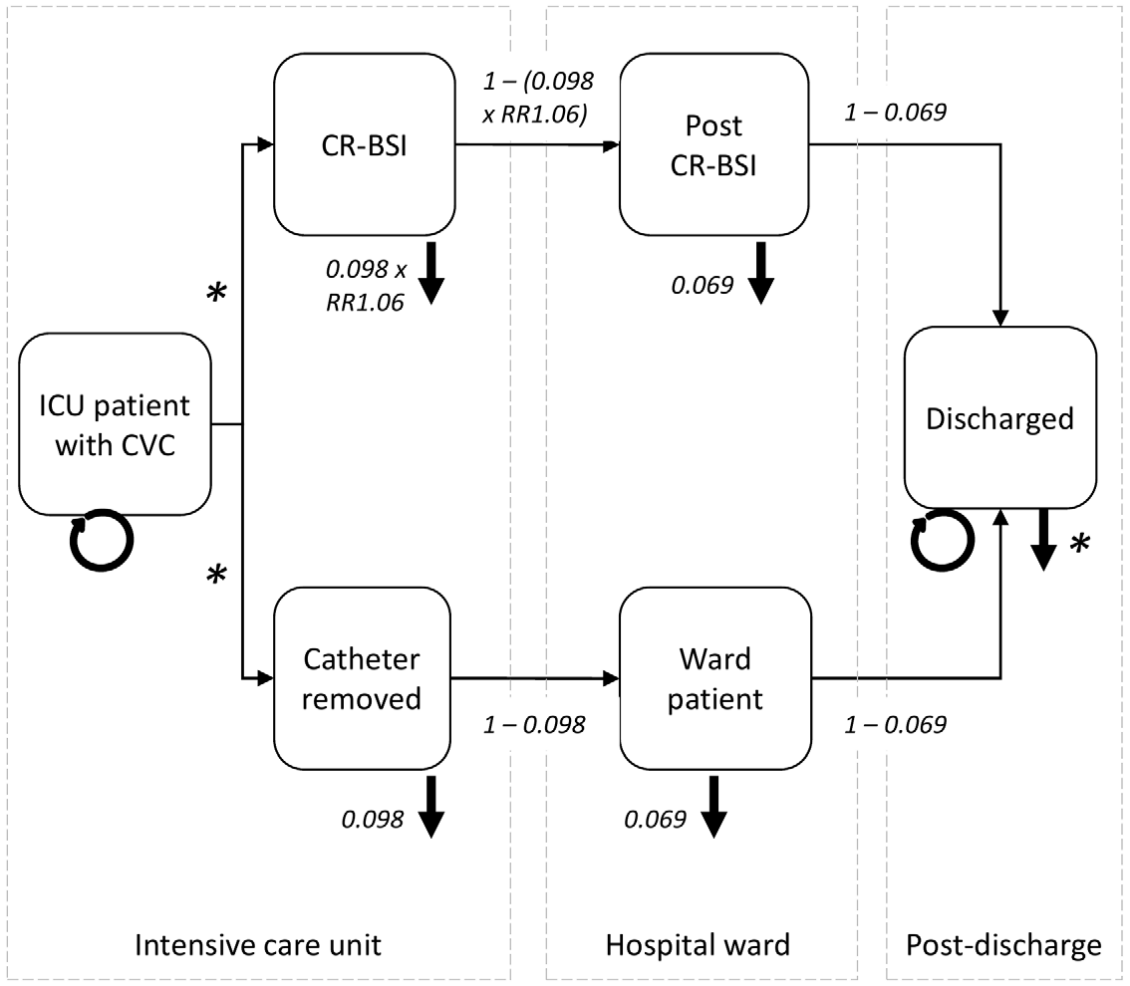
Markov model used for the economic evaluation. Arrows represent possible pathways for patient movement through the Markov model, circular arrows indicate the patient can remain in their current health state for subsequent cycles of the model, the small downward arrows from each health state represent mortality. Transition probabilities are shown. Where * is used, transition probabilities vary over time. See Table One for more details.

The context for the evaluation was a Level III adult intensive care unit (ICU) in an Australian public teaching hospital. Clinical and economic events under a healthcare perspective were identified in conjunction with intensive care clinicians and organized into Markov states (Figure 1) to create an economic decision model. This is the same model that has been used to evaluate the cost-effectiveness of A-CVCs in this context [12]. All models were built in Microsoft Excel.

Patients were assumed to receive a CVC on entry to ICU. Over subsequent cycles the catheter was either retained, removed as no longer necessary, or removed due to development of CR-BSI, which was defined according to the clinical definition used by the Centers for Disease Control and Prevention [6]. Patients faced an underlying risk of mortality representative of being in ICU and an elevated risk should they develop CR-BSI. The surviving cohort was followed for the remainder of their lifetime. We did not model catheter colonization as this event alone carries no health or economic consequences. We assumed all catheters were inserted or removed within the ICU and that there were no multiple catheterizations. The consequences of CR-BSI were assumed to be independent of patient age or disease severity and causative micro-organisms. Economic costs were measured in 2006 Australian dollars. Health outcomes were measured in quality-adjusted life years (QALYs). A discount rate of 3% was applied to costs and QALYs occurring in future time periods [16].

Data used in the model, its source and the level of this evidence as judged by the modified version of the hierarchy of data sources for economic evaluation [17] are shown in Table 1. Where possible, estimates for epidemiological parameters were obtained from the published literature using searches undertaken in Medline from January 1985 to January 2008. Cost data was sourced primarily from routine databases held by the Queensland State and Australian Federal healthcare systems.

**Table 1:** Parameters used in the model.

Parameters		Estimate	Source	Ref	Level of evidence
**Infection related events**					
Daily probability CR-BSI	Day 1–5	0.004	database & Q–E study	[22] [21]	1
	Day 6–15	0.009			
	Day 16–30	0.020			
RR mortality CR-BSI		1.06	Q–E study	[1]	2
Daily probability catheter removal		*time varying*	Q–E study	[21]	2
**Baseline mortality (probabilities)**					
ICU		0.098	data linkage study	[24]	2
Hospital		0.069			
Annual post-discharge	Year 1	0.050			
	Year 2–3	0.027			
	Year 4–5	0.028			
	Year 6–10	0.037			
	Year 11–15	0.042			
Underlying annual mortality	45–64 yrs	0.004	national statistics	[25]	1
	65–84 yrs	0.030			
	85+ yrs	0.140			
**Utilities**					
ICU		0.66	elicitation study (EQ-5D)	[26]	3
Population norms	50–59 yrs	0.80	population based survey	[28]	3
	60–69 yrs	0.79			
	70–79 yrs	0.75			
	80+ yrs	0.66			
**Cost of CR-BSI**					
Extra days ICU		2.41	Q–E study	[23]	2
ICU bed-day (2006 AUD)		3,021	costing study	[30]	4
Extra days hospital		7.54	Q–E study	[23]	2
Hospital bed-day (2006 AUD)		843	prior economic evaluation	[31]	3
Diagnostics CR-BSI (2006 AUD)		101.7	health system database	-	1
Treatment CR-BSI (2006 AUD)		591.3			
**Effectiveness infection control (RR)**					
CH/SSD catheter		0.66	meta-analysis	[12]	1+
MR catheter		0.39			
Bundle		0.34	Q–E study	[33]	2
**Additional cost infection control (per catheter; 2006 AUD)**					
CH/SSD catheter		11.64	health system database	-	1
MR catheter		59.36			
Bundle		*unknown*	-	-	6

Abbreviations: s.e. standard error; CR-BSI catheter related bloodstream infection; ICU intensive care unit; Q–E quasi-experimental; AUD Australian dollar; ec. eval'n economic evaluation; CH/SSD chlorhexidine & silver sulfadiazine; (int/ext) internal & external coating; SPC silver, platinum & carbon; MR minocycline & rifampicin; popul'n population; per comm. personal communication; A-CVC antimicrobial central venous catheter; RR relative risk.

Based on a four year dataset of 11,790 ICU admissions we assumed that 17% would receive a CVC (Mullaney D. Methods and preliminary results for a data linkage project to determine long-term survival after intensive care unit admission [abstract]; 2008; Second International Conference on Quality Audit and Outcomes Research in Intensive Care; Christchurch, New Zealand). Our catheterised cohort had a mean age 62.7 (s.d. 17.2) years, mean Acute Physiology and Chronic Health Evaluation II score 17.1 (s.d. 8) and were 65% male, equivalent to admissions reported to 46 publicly funded ICUs by the Australia New Zealand Intensive Care Society [18]. Baseline risk of ICU mortality was 9.8% and 16.1% by hospital discharge. Daily probabilities of catheter removal was estimated by fitting a Weibull distribution to data from an epidemiological study of 7,467 CVCs [19] whilst daily probability of CR-BSI was modelled as increasing, in stepwise increments, with duration of catheterisation [19]. This gave a probability of infection of 2.5% comparable to that observed in routine surveillance data collected from February 2001 to December 2005 in 21 medium-to-large public hospitals in Queensland, Australia [20].

Relative risk of hospital mortality associated with CR-BSI was estimated to be 1.06 [1]. Given a 9.8% baseline risk this corresponds to an absolute increase in mortality just under 1%. Excess length of stay due to infection was estimated at 2.4 ICU and 7.5 general ward days [21]. Annual mortality rates for fifteen years post-ICU discharge were taken from a data linkage study [22] that followed over 10,000 Australian ICU patients. Subsequent life expectancy was based on Australian Institute of Health and Welfare published age-specific mortality rates [23]. Preference based utility weights from a study [24] with participant demographics similar to our cohort were assigned to cycles spent in the ICU and 6 months immediately post-discharge. No further quality of life decrement was attributed to CR-BSI. As we did not consider side effects for the interventions (negative or positive) we did not incorporate these into the utility weightings. Although evidence suggests that quality of life may be reduced in some survivors for a longer period post-discharge [25], information on this was unavailable for our population, so, to be conservative, life expectancy for those surviving beyond this period were adjusted using Australian population quality of life norms [26].

All costs were valued at 2006 prices, using the Bureau of Labor Statistics Consumer Price Index [27] to adjust where necessary. Consumable costs in the evaluation included the price of the catheters, CR-BSI diagnosis costs (one catheter tip and two blood cultures) and treatment costs. Treatment costs were a weighted average of the cost of standard regimens for causative organisms observed within the surveillance system: two weeks Vancomycin, ten days Ticarcillin/clavulanate, four weeks Fluconazole. The cost for all consumables reflected prices faced by Queensland Health decision-makers. The economic value of bed-days released by the prevention of CR-BSI was estimated based on their likely value to a Queensland Health decision-maker. Values for an ICU bed-day were obtained from a detailed costing study of an Australian ICU [28] and for a general ward bed-day from an earlier economic evaluation which considered spending patterns for Australian public hospital services [29]. These estimates of $3,021 and $843 represent short-run average costs calculated by dividing total costs (i.e. fixed and variable costs) by the total bed days for a 12 month budget period. It is unknown whether these accounting based costs approximate the economic opportunity cost of the marginal bed day.

Estimates for the effectiveness of each type of commercially available A-CVC relative to uncoated catheters were taken from the results of a meta-analysis which provided separate estimates of effectiveness for each catheter type [11]. The additional cost of each antimicrobial catheter type (relative to uncoated catheters) was based on their cost on Standard Offer Arrangement with Queensland Health. We assumed that there would be no other costs associated with the introduction of antimicrobial catheters as the mechanisms for procurement, storage and usage of these catheters are identical to those used for uncoated catheters. To estimate the total cost of each type of A-CVC over 18 months, we estimated the total number of catheters that would be used during this time, based on data reported to the Australia and New Zealand Intensive Care Society 2003-05 resources and activity report [18]; 46 of 47 eligible Australian Level III public ICUs contributed data, reporting a total of 53,470 adult Level III ICU admissions annually. Assuming 17% would receive a catheter (Mullaney D. Methods and preliminary results for a data linkage project to determine long-term survival after intensive care unit admission [abstract]; 2008; Second International Conference on Quality Audit and Outcomes Research in Intensive Care; Christchurch, New Zealand), and making the simplifying assumption that patients receive only one catheter, we predicted 13,635 catheterisations over an 18 month period.

### Knowledge about the effectiveness and cost of a catheter care bundle

A variety of bundled behavioural interventions to prevent CR-BSI have been evaluated and reported in the scientific literature. The quality of many of these studies is uncertain [30]. For this evaluation we used estimates of the effectiveness of a bundle from the study by Pronovost *et. al.* [31]. The intervention in this study forms the basis for the catheter care and management bundle advocated by the Institute for Health Improvement 100k Lives Campaign [3] which is now being recommended for use in Australia. The bundle was comprised of five elements: use of optimal hand hygiene, chlorhexidine skin antisepsis, maximal barrier precautions for catheter insertion, choice of optimal insertion site, and prompt catheter removal and kits containing all the equipment recommended for catheter insertion were created. Implementation included programs to educate staff about clinical leadership, and infection risk as well as the bundle. The intervention reduced the rate of CR-BSI from 7.7 to 1.4 per 1,000 line days over an 18 month period; a reduction in relative risk of 0.34 (95% CI 0.23, 0.50).

The costs of implementing the bundle fall into two groups. First are those related to the five components. A list of the resources required to implement these is given in Table 2. It is estimated that the cost of these resources to Queensland Health decision-makers would be minimal. The second group of resources are those related to the monitoring, education and leadership activities. Table 2 summarises the resources associated with these activities based on descriptions provided across six publications produced from the Pronovost *et. al.* study [9], [31], [32], [33], [34], [35], but any measurement and valuation of the resources required to implement the bundle in an Australian context will be complex. Some of the resources used in implementing the Pronovost *et. al.* bundle were shared with existing quality improvement activities and the concurrent implementation of a comprehensive unit-based safety program, moreover, the units received substantial administrative support and mentoring from both the Michigan Health & Hospital Association Keystone Center for Patient Safety & Quality and Johns Hopkins University [35]. It is not clear if this kind of infrastructure or support would be available in Australia and what the costs of accessing it may be. A final group of costs relating to the bundle are those incurred by the national campaigns such as the 100k Lives campaign [3] which promote the use of these strategies. As the campaigns have already run, they represent sunk costs which cannot be recovered. As such they are not relevant to future decision-makers and are excluded from this evaluation.

**Table 2:** Resources potentially required to implement a catheter care bundle.

Activity	Resources	
	Unit	Frequency
**Catheter care bundle components**		
Components		
Appropriate hand hygiene	30 seconds	per catheter
Maximal sterile barrier precautions at insertion		
mask, gloves, gown, cap, large drape	1 set	per catheter
preparation of precautions	20 minutes	per catheter
Chlorhexidine skin preparation	7 day supply	per catheter
Subclavian vein placement preferred	no additional activity	
Prompt removal of catheters	no additional activity	
Central line supply care		
Stocking of cart	no data	every 4 hours
Use of cart	time saving	per catheter
**Education & monitoring activities**		
Education		
Nurse lectures	40 minutes	16 lectures
Physician lectures	40 minutes	5 lectures
Web-based physician training module	no data on duration or attendees	no data on repetition
Nurse orientation module	no data on duration or attendees	no data on repetition
Posters & factsheets	no data on numbers	no data numbers
Checklist for insertion		
Training on use	no data on duration or attendees	no data on repetition
Use of checklist	2 minutes	per catheter
Feedback on performance	no data on resources	no data on repetition
Collation & feedback of infection rates	no data on resources	no data on repetition
**Mentoring & leadership activities**		
Keystone project support*		
Conference calls from Keystone	no data on duration or attendees	2 per month
Conference calls from Hopkins	no data on duration or attendees	1 per month
Statewide meetings	no data on duration or attendees	2 per year
Participant website	no data on resources	no data on updates
Bimonthly e-newsletter	no data on resources	2 per month
Institution visits	no data on resources	no data on repetition
Key personnel (average four) per hospital		
Physician leader	4–8 hours	per week
Nurse leader	4–8 hours	per week
Senior executive	4–8 hours	per week
Staff nurse/infection control practitioner/pharmacist	4–8 hours	per week
Program activities		
Education session & safety survey	40 minutes, all staff	once
Senior executive meetings	no data on duration or attendees	1 per month
Daily goals sheet for communication	no data on resources	3 per day
Nurse pre-discharge medication review	no data on resources	per admission
Web-based error reporting system	no data on resources	no data on maintenance

*Keystone funded to $15 million to undertake multiple projects not just a catheter care bundle.

Resources were identified based on the following publications:

Pronovost PJ, Berenholtz S, Dorman T, Lipsett PA, Simmonds T, Haraden C: Improving communication in the ICU using daily goals. J Crit Care 2003, 18:71–75.

Pronovost PJ, Weast B, Bishop K, Paine L, Griffith R, Rosenstein B, Kidwell RP, Haller KB, Davis R: Senior executive adopt-a-work unit: a model for safety improvement. Jt Comm J Qual Patient Saf 2004, 30:59–68.

Pronovost PJ, Goeschel C: Improving ICU care: it takes a team. Healthc Exec 2005, 20:15–22.

Pronovost PJ, Weast B, Rosenstein B, Sexton B, Holzmueller CG, Paine L, Davis R, Rubin HR: Implementing and validating a comprehensive unit-based safety program. Journal of Patient Safety 2005, 1:33–40.

Pronovost PJ, Needham DM, Berenholtz S, Sinopoli D, Chu H, Cosgrove SE, Sexton B, Hyzy R, Welsh R, Roth G, Bander J, Kepros J, Goeschel C: An intervention to decrease catheter-related bloodstream infections in the ICU. N Engl J Med 2006, 355:2725–2732.

Berenholtz SM, Pronovost PJ, Lipsett PA, Hobson D, Earsing K, Farley JE, Milanovich S, Garrett-Mayer E, Winters BD, Rubin HR, Dorman T, Perl TM: Eliminating catheter-related bloodstream infections in the intensive care unit. Crit Care Med 2004, 32:2014–2020.

### Evaluating the decision

The cost of implementing a bundle in Australia is unknown. As such deterministic threshold analyses [36] were used to evaluate the decision with all parameters characterised as point estimates. The maximum cost for the bundle was identified at which it would remain cost-effective assuming it would reduce risk of CR-BSI by RR CR-BSI 0.34. Given uncertainty about whether the same reduction in risk would be seen in Australia, we also estimated the cost-effectiveness of the bundle for different combinations of effectiveness and cost. For each combination the bundle was identified as being either cost-saving, cost-effective (i.e. the cost per QALY was less that AUD $64,000 [37]) or not an efficient investment (i.e. the cost per QALY was more than AUD $64,000). Results were plotted on a graph of ‘bundle cost’ against ‘bundle effectiveness’, and the maximum cost at which the bundle remained cost-effective was identified for each level of effectiveness. The threshold costs estimated represent total Australia wide implementation costs over an 18 month time period.

Recognising that clinicians hold different preferences about the use of A-CVCs, the threshold analysis was undertaken for three separate comparisons. First a pair-wise comparison was made between current practice (where uncoated catheters and a non-bundled approach to catheter management are used) and the bundle. This comparison is relevant for decision makers who reject the use of all A-CVCs. Then a three-way comparison was made: current practice v. bundle v. CH/SSD catheters. This comparison is relevant to decision makers prepared to use antiseptic catheters but who reject the use of antibiotic devices. Finally, a four-way comparison is made of current practice v. bundle v. CH/SSD catheters v. MR catheters. This represents the preference of a decision maker happy to consider use of all technologies.

The results of the baseline analyses enable the decision-maker to judge whether the bundle is likely to be cost-effective given their belief about the likely cost and effectiveness of a proposed bundled intervention, and their current preferences regarding the use of A-CVCs in infection control. They assume that the decision maker values the full costs of supplying bed days and the full economic benefit of generating QALYs. To explore the modelling further we undertook three sensitivity analyses for each comparison. These sensitivity analyses represent alternate perspectives for decision making as valuations placed on QALYs and bed-days by decision makers may vary.

First, we recalculated the cost-effectiveness threshold under the assumption that the decision maker was not willing to pay for QALYs/health benefits and was solely interested in the option which minimised total costs. Second, we recalculated the cost-effectiveness threshold assuming the decision maker was willing to pay for health benefits (up to $64,000 per QALY) but were only interested in avoiding the variable (or consumable) costs associated with the extra length of stay and did not value the opportunity cost of these bed-days (that is, the extra unit capacity obtained). To do this the valuation for marginal bed-days was changed to reflect the daily variable costs only, reported in a similar patient population [38]; this assumes it would be later (less costly) days released by preventing infection and gives estimates of AUD $362 ICU and AUD $101 general ward. Third, we recalculated the cost-effectiveness threshold assuming that the decision-maker was not willing to pay for either health benefits or the extra unit capacity released. The interpretation of this scenario is that decision makers are interested solely in the cash-savings (variable costs avoided) to the unit.

## Results

**Figure 2:**
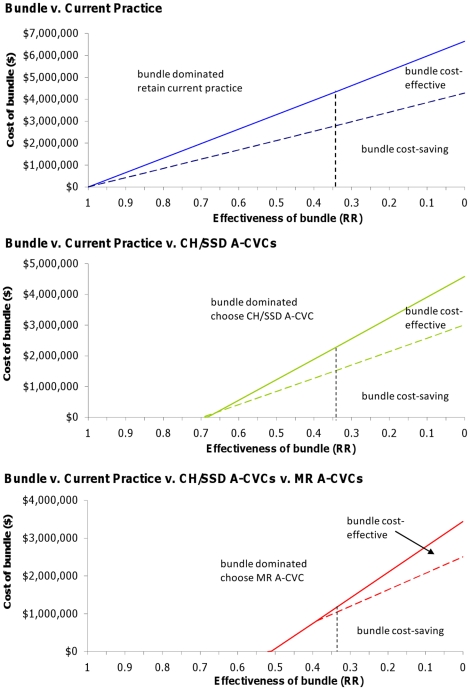
Cost and effectiveness thresholds for a catheter care bundle versus alternative infection control interventions.

Figure 2 shows the cost and effectiveness thresholds for the catheter care bundle compared to uncoated, CH/SSD and MR catheters.

The area marked “bundle cost-saving” on each graph indicates combinations of cost and effectiveness at which the bundle reduces total costs and delivers extra health benefits relative to the comparator. The area marked “bundle cost-effective” shows combinations of cost and effectiveness for the bundle at which total costs increase but the cost per QALY is less than $64,000. Any combination of cost and effectiveness which places the bundle in the region marked “dominated” indicates the bundle is not an efficient investment and the decision-maker should choose the comparator indicated.

The comparator that should be chosen differs depending on the preference of the decision maker. Where A-CVCs are not included in the decision, the preferred option where the bundle is dominated is to remain with current practice. Where the decision maker is prepared to use A-CVCs these are the preferred option should the bundle be dominated as they themselves are cost-effective relative to remaining with current practice [12]. As the MR catheter is cost-saving relative to CH/SSD catheters these are the preferred option in the four-way comparison if the bundle is dominated.

For all comparisons, the more effective the bundle is, the higher the cost threshold at which it remains an efficient investment. The baseline results summarised in Table 3 show the cost at which the bundle remains cost-effective, assuming, for the Australian context, that it will deliver a reduction in risk of CR-BSI equivalent to that seen in Pronovost et al. (RR CR-BSI  = 0.34) [31]. If this effective, it is cost-effective up to a total nationwide 18 month implementation cost of $4,349,730 when compared only to current practice, $2,287,400 when CH/SSD catheters are included in the decision and $1,144,465 when MR catheters are considered a relevant alternative.

**Table 3:** Cost and effectiveness thresholds for a catheter care bundle under different perspectives.

Scenario		Baseline	No value given to QALYs	No value given to extra unit capacity	Interested only in cash-savings
*Willingness to pay for a QALY*		*$64,000*	*$0*	*$64,000*	*$0*
*Value for an ICU bed-day*		*$3021*	*$3021*	*$362*	*$362*
*Value for a ward bed-day*		*$843*	*$843*	*$101*	*$101*

Comparisons					
Bundle v. current practice					
	choice if bundle dominated	Current practice	Current practice	Current practice	Current practice
	minimum RR for bundle to dominate	0.999	0.999	0.999	0.999
	cost threshold at RR = 0.34 (estimate from Pronovost et al.)	$4,349,730	$2,801,690	$2,020,600	$472,550
	cost threshold at RR = 0.001 (theoretical maximum)	$6,646,520	$4,285,050	$3,087,550	$722,100

Bundle v. CH/SSD catheters v. current practice					
	choice if bundle dominated	CH/SSD cost-saving	CH/SSD cost-saving	CH/SSD cost-saving	CH/SSD cost-saving
	minimum RR for bundle to dominate	0.684	0.697	0.712	0.883
	cost threshold at RR = 0.34 (estimate from Pronovost et al.)	$2,287,400	$1,529,810	$1,147,560	$389,970
	cost threshold at RR = 0.001 (theoretical maximum)	$4,584,200	$3,013,160	$2,214,500	$639,500

Bundle v. MR catheters v. CH/SSD catheters v. current practice					
	choice if bundle dominated	MR cost-saving	MR cost-saving	MR ICER $16,717/qaly	CH/SSD cost-saving
	minimum RR for bundle to dominate	0.511	0.579	0.652	0.883
	cost threshold at RR = 0.34 (estimate from Pronovost et al.)	$1,144,465	$1,025,205	$965,030	$389,970
	cost threshold at RR = 0.001 (theoretical maximum)	$3,441,280	$2,508,550	$2,031,980	$639,500

All cost thresholds represent nationwide implementation costs over an 18mth period. Costs per ICU can be obtained by dividing each figure by 46 e.g. given RR  = 0.34 the cost threshold per ICU for the bundle relative to no intervention equals $4,349,730/46 = $94,559.

**Figure 3:**
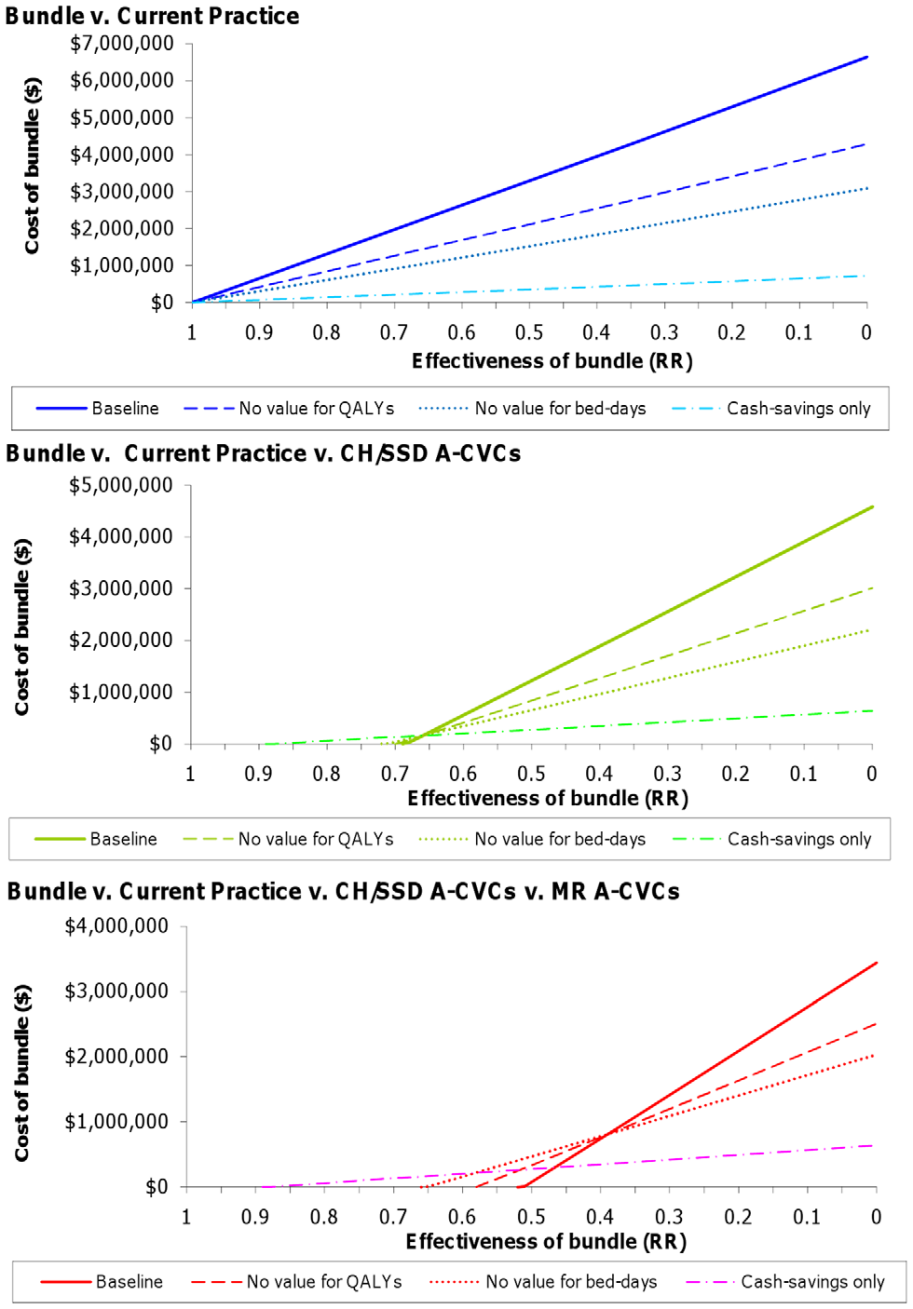
Cost and effectiveness thresholds for a catheter care bundle under different decision making perspectives.

It is important to note that when both CH/SSD and MR catheters are being considered, the MR catheters are the preferred option where the bundle is dominated for all scenarios except where health benefits are valued at zero and bed-days only at the value of variable costs. Under this scenario where the bundle is dominated the MR catheters are not cost-effective as the cost per QALY exceeds $64,000 and it is the CH/SSD catheters that are preferred, hence the shift in the threshold seen in Figure 3c.

Table 3 also shows the maximum cost for at which the bundle would remain cost-effective assuming it would be able to effectively eliminate infection (RR CR-BSI  = 0.001). Under this assumption nationwide implementation costs would have to be under $6.6 million when A-CVCs are not considered a relevant option, $4.5 million when CH/SSD catheters are included in the decision and $3.4 million when MR catheters are included. This represents a theoretical upper limit on the cost of the bundle. Should implementation costs exceed this threshold the bundle cannot represent an efficient use of resources as the cost per QALY will always exceed $64,000.

The minimum level of effectiveness at which the bundle would remain cost-effective assuming negligible implementation costs ($1) is given in Table 3. Although this assumption is unrealistic as implementation costs are unlikely to be this low, this represents a theoretical lower limit on effectiveness below which expenditure on the bundle will always be an inefficient use of resources. When compared to current practice and excluding A-CVCs from the decision, the bundle has the potential to be cost-effective as long as it is effective and prevents infections (i.e. RR ≤0.999) However, when A-CVCs are introduced to the decision, for the bundle to be cost-effective even with negligible implementation costs, it must achieve a minimum level of effectiveness of RR  = 0.684 when CH/SSD catheters are an option and RR = 0.511 when MR A-CVCs are also being considered. This reflects the fact that the A-CVCs are themselves effective and cost-saving in reducing risk of CR-BSI.

The results of the sensitivity analysis are shown in Figure 3. If decision makers are not willing to pay for QALYs and just want to minimise total costs, the cost threshold at which the bundle is a good investment is reduced by around a third relative to the baseline analysis for all hypothetical levels of effectiveness. If decision makers place no value on the extra capacity released by infection control (valuing released bed-days at only the variable costs saved) then the cost threshold at which the bundle is cost-effective is halved relative to the baseline analysis. If decision-makers value neither QALYs nor the opportunity cost of bed-days, and are only interested in obtaining cash-savings for the unit, the cost-threshold is reduced by two-thirds for any given level of effectiveness relative to the baseline analysis. The precise thresholds for each comparison are given in Table 3.

## Discussion

Because clinicians have different preferences for using A-CVCs we compared the cost-effectiveness of a catheter care bundle to a baseline (representing use of uncoated catheters and a non-bundled approach to catheter management) and then to both CH/SSD and MR catheters in separate analyses. This enables the decision-maker to judge the likely cost-effectiveness of a bundled approach given their preference regarding the use of these devices.

The study by Pronovost et al. estimated a bundled approach was associated with a relative risk of 0.34 (95% CI 0.23, 0.50) [31]. At this level of effectiveness, if clinicians are prepared to use MR catheters in their clinical practice, the bundle is cost-effective if nationwide implementation costs over an 18 month period are below $1.1 million nationally. If costs exceed this then they should invest in MR A-CVCs as these are cost-effective relative to current practice. Given that this analysis was based on 46 Level III ICUs, this represents a maximum 18 month budget per ICU of $24,880. The uptake of A-CVCs in Australia is very limited [15] despite evidence about the effectiveness and cost-effectiveness of these devices. This may be related to clinician concerns about the use of these devices that is not captured in the economic evidence [12]. If A-CVCs are not a relevant option then a bundle must cost less than $4.3 million nationwide, which represents a budget of $94,559 per ICU. If costs exceed this threshold then the units should remain with current practice.

The economic outcomes from infection control are summarised in terms of QALYs and total costs. The main component of total costs is the opportunity cost assigned to each bed-day released by infection control [39], which is its value in being able to use it to treat another patient. Our baseline analysis assumed that decision-makers were prepared to pay $64,000 per QALY and $1032 per ICU bed-day. However, these figures represent preferences which may vary according to the perspective of the decision-maker. Our baseline valuations represent a broad healthcare perspective. A unit manager operating on a fixed budget may hold different values for these outcomes. The results of our sensitivity analysis show that the cost threshold at which the bundle remains the preferred choice drops by a third if no value is placed on QALYs and halves if no value is given to the opportunity cost of bed-days. Infection control is often referred to as being “cost-saving”. Our final sensitivity analysis highlights how this term should not be confused with “cash-saving”. If a decision-maker is concerned only with reducing unit cash expenditures, and places no economic value on QALYs or extra bed days (i.e. the capacity to treat new patients), then the cost threshold for the bundle relative to no intervention (given a RR  = 0.34) drops from $4.3 million to $472,550.

Infection control interventions are often cost-saving, but this term can be somewhat misleading, as most infection control interventions are unlikely to produce cash savings for the unit into which they are introduced and, in fact, the opposite may occur. The cost-savings these infection control interventions achieve are better thought of as opportunity costs avoided, the main component of which is the value assigned to the bed-days released by infection control. Most of the costs of hospital care (84%) are fixed in the short term [40], and, as the fixed running costs of the ICU cannot be recovered, they will not alter with changes to rates of infection [41], [42]. Units which expend more of their funds on infection control are unlikely to see cash returns on this investment. Although the extra capacity enables the treatment of more patients, the initial review, diagnosis and establishment of another patient is costly in terms of consumables and staff time which will drive up variable costs. Overall, the healthcare system as a whole will become more efficient, and average cost per patient will fall, but this increase in the variable costs needed to treat these extra patients may result in an increase to total costs in the healthcare system [5]. Focusing on the economies resulting from improving the use of fixed costs, rather than focusing on achieving a reduction in expenditure is likely to be a better approach to justifying and sustaining investment in infection control [43], but even so, decision-makers at both the local (unit) level and the level of the healthcare system, be it centralised or de-centralised, must be prepared to invest resources in infection control to see this efficiency improvement.

The likelihood of being able to implement a catheter care bundle in Australia for less than $4.3 million is unclear. It would depend upon the resources required for implementation (the main component of which is likely to be personnel and staff time), the degree to which these resources would be centralised rather than spread (and possibly duplicated) across individual States/Territories and/or hospital sites, the value of these resources and how often these costs would have to be incurred. A budget of $94,559 per ICU would pay for a coordinator in each site but probably little else. Moreover, although a $4.3 million dollar catheter care bundle initiative would be cost-effective, it would represent a substantial investment. For decision-makers facing finite budgets, any increases to present levels of funding for infection control activities will necessitate decisions about from which other area of healthcare these extra funds should be sourced. An investment of this magnitude would require careful consideration to ensure that other important, effective and efficient services were not displaced. Given the announcement of a $4 million investment in the National Hand Hygiene Initiative in May 2009, it may be that there will not be the funds to support another large scale initiative aimed at, what may be perceived to be, a very similar healthcare objective. On the other hand the development of a national infrastructure for rolling out infection control initiatives may drive the cost of subsequent initiatives down as spare capacity in existing personnel and resources could be utilised. This would provide further support for implementing a catheter care bundle.

There are a number of limitations to this analysis of the cost-effectiveness of a bundled behavioural intervention for preventing CR-BSI, and the use of this information for infection control decision making. Evidence for a bundled behavioural intervention to prevent CR-BSI suggests that this approach may be highly effective [30]. However, the evidence is undermined by problems in the design and conduct of the trials, and as all studies used slightly different variants of the bundle it is difficult to predict whether a comparable reduction in risk would be achievable in Australia. We were unable to estimate the cost of this type of intervention in an Australian context because it was unclear what the full resource requirements are for the implementation of bundle, and what the value of these would be. As such we undertook a threshold analysis to identify the cost and effectiveness thresholds at which a hypothetical bundle would be cost-effective relative to no investment in infection control and the thresholds at which it would dominate the use of A-CVCs. This approach places the onus on others to make a judgement about levels of cost and effectiveness of the bundle which limits its ability to guide efficient decision making. A high quality study that estimates the costs of implementing a bundle would increase the usefulness of this modelling.

As the estimates of effectiveness for all interventions are measured in relative units, the specific estimates of cost-effectiveness for the A-CVCs and the cost and effectiveness thresholds obtained for the bundle relate specifically to a baseline infection rate of 2.5%. For decision-makers in settings where the infection rate differs significantly from this value, care should be taken in interpreting these results and caution used in basing decisions on specific thresholds. Although this difference may not change the relative ordering of the interventions, where initial rates of infection are higher the precise cost threshold at which the bundle is cost-effective for any given level of effectiveness will be higher, and where infection rates are lower, the threshold will be lower.

It is also likely given the broad range of outcomes decision makers consider when deciding on the adoption of a new technology [44], [45] that not all relevant costs and benefits have been captured in this evaluation. There are a number of potential negative externalities that can result from the use of A-CVCs [13], [14], and several positive ones that may be delivered by a catheter care bundle [9], [34] that have not been included. This evaluation did not include less tangible benefits to reduced infection rates, such as increases to clinical morale and public confidence in the healthcare system demonstrated by the national campaigns to reduce rates of CR-BSI [3] and forming part of the rationale for the introduction of the Deficit Reduction Act in the United States [4]. Valuing these outcomes would be difficult to achieve and these concerns may be best considered in a decision-making framework alongside, rather than subsumed into, the economic evidence. Excluding these costs and benefits from the analysis does not result in them being excluded from the adoption decision. Instead, it leaves decision-makers to consider them implicitly, creating the potential for these concerns to be assigned arbitrary values based on the potential for bad press or political backlash. Their inclusion would improve the representation of the economics of preventing infection and more accurately reflect the trade-offs being made by decision-maker which may help prevent this reactionary approach to decision-making.

### Conclusion

Estimates of the cost of a catheter care bundle should include the costs of monitoring, education and clinical leadership activities, as well as the cost of the consumables used in the care practices included in the bundle. If clinicians are prepared to use antimicrobial catheters in their clinical practice, a bundle which achieves a relative risk of catheter-related bloodstream infection of 0.34 is cost-effective if nationwide implementation costs over an 18 month period are below $1.1 million nationally, a budget of $24,880 per intensive care unit. If costs exceed this then minocycline and rifampicin-impregnated catheters should be used. If antimicrobial catheters are not a relevant option then a bundle must cost less than $4.3 million nationally, which represents a budget of $94,559 per unit. If costs exceed this threshold then the units should remain with current practice, defined here as uncoated catheters and a non-bundled approach to catheter management. These thresholds assume that decision makers are willing to pay for both health benefits obtained and bed-days released by infection control. If no economic value is placed on the bed-days released by infection control then these cost thresholds are halved, whilst if no economic value is placed either the bed-days or the health benefits gained through preventing infection, these cost-thresholds are reduced by two-thirds. Rather than anticipating cash-savings decision makers should be prepared to invest resources in infection control to gain efficiency improvements in the intensive care unit.
